# Relation Between Awe and Environmentalism: The Role of Social Dominance Orientation

**DOI:** 10.3389/fpsyg.2018.02367

**Published:** 2018-12-03

**Authors:** Huanhuan Zhao, Heyun Zhang, Yan Xu, Jiamei Lu, Wen He

**Affiliations:** ^1^Department of Psychology, Shanghai Normal University, Shanghai, China; ^2^School of Social Administration, Shanghai University of Political Science and Law, Shanghai, China; ^3^Beijing Key Laboratory of Applied Experimental Psychology, National Demonstration Center for Experimental Psychology Education (Beijing Normal University), Faculty of Psychology, Beijing Normal University, Beijing, China

**Keywords:** awe, self-transcendent emotion, social dominance orientation, environmentalism, environmental engagement

## Abstract

The present study attempts to explore the effect of awe on environmentalism and the mediating role of social dominance orientation in generating this effect. In Study 1, a series of questionnaires were used to investigate the correlation among trait awe, social dominance orientation, and ecological behavior. Results demonstrated that, while trait awe was positively correlated with ecological behavior, it was partially mediated by social dominance orientation. In follow-up studies, two priming experiments were conducted to test the causal relationship and the psychological mechanisms between awe and environmentalism. Results revealed that inductions of awe (relative to various control states) decreased participants’ social dominance orientation, which in turn partially enhanced their willingness to make personal sacrifices for the environment (Study 2), and intentions to engage in pro-environmental behavior (Study 3). This study not only corroborates the critical role of awe in promoting environmentalism, but also highlights the importance of social dominance orientation in explaining why awe increases environmentalism. Implications and future directions were also discussed.

## Introduction

Environmental deterioration is one of the most troubling problems of humanity. Various environmental problems, such as air pollution, water shortage, land degradation, global warming and biodiversity decline, pose severe threats to humankind’s sustainable development. Many of these problems are due to human behavior (Vlek and Steg, 2007; Steg and Vlek, 2009). Therefore, changing people’s environmentally harmful behavior and exploring methods to encourage increased engagement in activities that protect the environment have become vital tasks.

Psychological science helps solve current environmental problems by identifying the main drivers and barriers of environmental protection behaviors (e.g., Onwezen et al., 2013; Gifford, 2014; De Leeuw et al., 2015; Zelenski et al., 2015). For example, recent studies have shown that self-conscious emotions (e.g., pride, guilt) are important factors that can potentially influence environmental behaviors (Ferguson and Branscombe, 2010; Harth et al., 2013; Onwezen et al., 2013; Bissing-Olson et al., 2016). However, the role that awe, an important self-transcendent emotion, plays in environmental behavior change is unclear. A Chinese saying states that “keep awe in mind, and you will stay out of improper behavior.” Awe dissuades people from focusing on immediate self-interests and encourages them to consider the welfare of others and that of the broader external environment (Rudd et al., 2012; Piff et al., 2015; Prade and Saroglou, 2016; Bai et al., 2017). A growing body of evidence suggests that awe considerably influences the promotion of prosocial behaviors (Rudd et al., 2012; Piff et al., 2015; Prade and Saroglou, 2016). We therefore posit that this relationship may be extended to environmentalism.

Environmentalism is widely defined as concern for the environment and support for environment-friendly behaviors, intentions, and attitudes (Milfont et al., 2013). Many researchers have discussed environmentalism as a multifaceted construct (Jia et al., 2015; Milfont et al., 2017; Stanley et al., 2017). For example, Milfont et al. (2017) proposed to study environmentalism by investigating environmental citizenship actions, pro-environmental behaviors, and donations to environmental organizations. Jia et al. (2015) measured environmentalism through environmental involvement, environmental identity, and environmental attitudes. In the present study, we utilized three indicators, namely, ecological behavior, environmental sacrifice, and pro-environmental intention, to assess the multifaceted construct of environmentalism. Research has indicated that environmentalists possess a propensity to act pro-socially (Kaiser and Byrka, 2011). Thus, the purpose of this study is to elucidate the effect of awe on environmentalism. In other words, the current study aims to determine whether awe influences environmentalism and the possible psychological mechanism that underlies this relationship.

### Awe and Environmentalism

Awe is an emotion that arises when people encounter something so strikingly vast that it defies their current knowledge structures and provokes a need to update their mental schemas (Keltner and Haidt, 2003). Awe involves positively valenced feelings, such as wonder, amazement, appreciation, and admiration (Keltner and Haidt, 2003; Piff et al., 2015). Although awe experiences are tinged with fear, awe is typically considered a positive prosocial emotion (Gordon et al., 2016; Stellar et al., 2017). Various stimuli, such as natural wonders, beautiful art, extraordinary human accomplishments, intellectual epiphany, and religious experiences, can evoke the intense emotional response of awe (Keltner and Haidt, 2003; Shiota et al., 2007; Saroglou et al., 2008).

Awe is referred to a self-transcendent emotion, and it reflects self-transcendence values, encourages individuals to transcend their momentary desires, diminishes the emphasis on the individual self and self-interest, and shifts the attention of individuals toward the needs and concerns of others (Piff et al., 2015; Stellar et al., 2017, 2018). Research has indicated that self-transcendent emotions are other-oriented and work as powerful proximal determinants of prosocial action (Stellar et al., 2017). Ample evidence suggests that positive awe experiences in daily life and in the laboratory enhance the welfare of others and motivate people to engage in various forms of prosocial behaviors (Rudd et al., 2012; Piff et al., 2015; Prade and Saroglou, 2016). For example, in Rudd et al. (2012) study, participants who experienced awe were more willing to volunteer their time to help others compared with those who did not experience awe. Piff et al. (2015) conducted a series of studies and discovered that dispositional tendencies to experience awe were positively associated with increased generosity in an economic game, and experimentally inducing awe makes participants endorse ethical decision-making and helping behaviors and results in numerous prosocial values. In addition, some evidence shows that awe effectively reduces antisocial behaviors, such as aggressiveness (Yang et al., 2016). The subjective experience of awe is consistent with the notion of self-transcendence (Shiota et al., 2014). Prior research has demonstrated that the endorsement of self-transcendence values encourages people to engage in pro-environmental behaviors (Cheung et al., 2014; Jia et al., 2017). Therefore, given the preceding discussion and the generally prosocial nature of environmentalism, awe is expected to be positively related with environmentalism.

### The Mediating Role of Social Dominance Orientation

The reason a positive relationship may exist between awe and environmentalism remains unclear. In this study, we focus on the role of social dominance orientation in explaining the relationship between awe and environmentalism. Specifically, we suggest that awe may encourage people to engage in pro-environmental actions because it can reduce their dominance over nature.

The belief that humans can dominate over nature is at the heart of current environmental problems (Milfont et al., 2013, 2017). Human dominance over nature is conceptually related to social dominance theory, which focuses on individuals’ attitudes about hierarchical and unequal relations between groups in society (Pratto et al., 1994; Sidanius and Pratto, 1999). Social dominance orientation is the core individual-level variable in social dominance theory, which reflects individuals’ preference for group-based hierarchy and inequality (Pratto et al., 1994). Individuals with high social dominance orientation are concerned with group or interpersonal dominance rather than general or individual equality (Pratto et al., 1994; Son Hing et al., 2007). Research has shown that social dominance orientation is closely related to various group-based attitudes and behaviors (Sidanius et al., 1994; Kteily et al., 2011, 2012). For example, social dominance orientation can reduce generosity in allocating resources to outgroups (Sidanius et al., 1994) and increase prejudice and discrimination against ethnic and racial outgroups (Kteily et al., 2011).

Although the focus of social dominance orientation is on a generalized orientation toward unequal and dominant/subordinate relations between humans, previous research has indicated that the theoretical scope of social dominance theory can be extended to understand person–environment relations (Milfont et al., 2013, 2017; Milfont and Sibley, 2014; Panno et al., 2017; Carrus et al., 2018). That is, the preference for hierarchy and inequality in the social world can translate into the preference for hierarchy in the natural world, with humans hierarchically dominating over nature (Milfont et al., 2017). Existing literature has demonstrated that social dominance orientation is inimical to pro-environmental attitudes and behaviors (Milfont and Duckitt, 2010; Milfont et al., 2013; Panno et al., 2017; Stanley et al., 2017). Individuals with high social dominance orientation show less concern about environmental issues (Milfont et al., 2013), are less supportive of environmental policies (Pratto et al., 1994) and more supportive of environmental inequality (Jackson et al., 2013), prioritize business gains over environmental protection, and exploit the environment in unsustainable ways (Son Hing et al., 2007). The need to maintain and enforce group-based hierarchical social structures causes them to dominate over the environment (Milfont and Sibley, 2014). In summary, social dominance orientation is negatively associated with environmentalism.

We now discuss the relationship between awe and social dominance orientation. In our view, several reasons support the notion that awe may be negatively associated with social dominance orientation. Evidence shows that social dominance orientation is positively related to self-enhancement values, which concern the enrichment of the self through the obtainment of achievement, power, and pleasure, and negatively related to self-transcendence values, which transcend the focus on the self and prioritize the welfare of the society, the maintenance of peace, justness, and protection of nature (Duriez and van Hiel, 2002). Conversely, awe, a self-transcendent positive emotion, is negatively related with self-enhancement values (Boer and Fischer, 2013). Thus, a consistent pattern emerges in which self-enhancement values have opposite associations with awe and social dominance orientation.

Research has also shown that awe can increase humility and decrease entitlement (Piff et al., 2015; Stellar et al., 2018). People exposed to awe-inducing stimuli are likely to feel a sense of self-diminishment, insignificance, and smallness (Shiota et al., 2007; Van Cappellen and Saroglou, 2012; Piff et al., 2015; Bai et al., 2017; Stellar et al., 2018). Conversely, individuals high in social dominance orientation often attach more value to superiority and dominance than to egalitarianism, feel superior and exhibit less concern for others (Lippa and Arad, 1999; Duckitt, 2001; Son Hing et al., 2007; Ho et al., 2015). A final piece of evidence supporting the notion that awe and social dominance orientation can be negatively related originates from research on pro-sociality. As previously mentioned, awe has been proven to increase ethical decision making, generosity, helpfulness, and prosocial values (Piff et al., 2015; Prade and Saroglou, 2016). Social dominance orientation operates in the opposite direction. Research has demonstrated that social dominance orientation causes people to make unethical decisions and exploit others for self-interest gains (Son Hing et al., 2007; Milfont et al., 2013). Accordingly, we propose that awe is negatively associated with social dominance orientation, and individuals with high levels of awe are more likely to exhibit low levels of social dominance orientation.

Overall, based on this hypothesized relationship between awe and social dominance orientation, and together with prior research showing that social dominance orientation is negatively associated with environmentalism, we assume that social dominance orientation may play a mediating role in the relationship between awe and environmentalism.

### Overview of the Current Study

On the basis of the aforementioned arguments and evidence, we established the following hypotheses:

Hypothesis 1: Awe is positively associated with environmentalism; and
Hypothesis 2: Social dominance orientation mediates the relationship between awe and environmentalism.

We conducted three sub-studies in China to test Hypotheses 1 and 2. Different environmentalism measures were considered to provide a powerful test for the two hypotheses. In Study 1, questionnaires were used to determine whether trait tendencies to experience awe predict ecological behavior and to examine whether social dominance orientation is a potential mediator between them. In follow-up studies, different priming experiments, including narrative recall (Study 2) and watching compelling video clips (Study 3), were conducted to further determine whether participants’ experience of awe increases their willingness to sacrifice for the environment (Study 2) and intentions to engage in pro-environmental behavior (Study 3) by reducing their social dominance orientation.

## Study 1 Correlational Research

Study 1 has two objectives. First, we explored whether trait awe is positively correlated with ecological behavior. Second, we tested whether social dominance orientation can mediate the effect of trait awe on ecological behavior.

### Methods

#### Participants

A total of approximately 600 Chinese adults were initially recruited from various organizations in China in a variety of industries including education, health care, business management, and information technology. Of the 556 participants who finally completed the questionnaires, 27 records were excluded from the analysis because of quality control checks (e.g., the same response was given across most of the survey), and the valid sample comprised 529 Chinese adults (304 female and 225 male; *M*_age_ = 29.55 years, *SD* = 8.89 years; age range: 18–56 years). Participants varied considerably in terms of education levels (11.90% with high school education or less, 18.90% with a college degree, 53.70% with a bachelor degree, and 15.50% with a post-graduate degree).

#### Procedure

All participants signed an informed consent form prior to the study, and then they were asked to fill out a series of self-report questionnaires within 25 min. Several questionnaires were translated from English to Chinese and back-translated for accuracy. After they completed the questionnaires, the participants were required to provide demographic information. Upon completion, they were thanked and debriefed. All procedures were reviewed and approved by the ethics board of Shanghai Normal University.

#### Measures

##### Trait awe

The dispositional positive emotion scale is widely used to assess trait awe (Shiota et al., 2006). In the current study, the Chinese version of the trait awe inventory was used to assess participants’ trait awe (Zhao et al., 2018, unpublished). This inventory consisted of 21 items rated on five-point Likert scale (1 = *strongly disagree*, 5 = *strongly agree*). One sample item is “I feel wonder almost every day.” Higher scores reflected that individual has a higher level of trait awe. The Cronbach’s α was 0.86.

##### Social dominance orientation

Social dominance orientation was measured using the eight-item version of the social dominance orientation scale (Ho et al., 2015). One sample item is “Some groups of people are simply inferior to other groups.” Each item was answered on a seven-point Likert scale (1 = *strongly disagree*, 7 = *strongly agree*), with higher scores indicating higher levels of social dominance orientation. The Cronbach’s α was 0.75.

##### Ecological behavior

The eight-item version of the ecological behavior scale was adopted to evaluate the frequency with which participants engaged in each of eight specific environmental activities, such as “looked for ways to reuse things” within the last year (Milfont and Duckitt, 2004). Items were rated on a five-point Likert scale (1 = *never*, 5 = *very often*), with higher scores reflecting more ecological behaviors. The Cronbach’s α was 0.81.

##### Control variables

We included gender, age, education, and social desirability as control variables that potentially influenced environmentalism (Dunlap et al., 2000; Zelezny et al., 2000; Kollmuss and Agyeman, 2002; Milfont and Duckitt, 2010), in order to isolate the independent effects of awe, social dominance orientation on environmentalism in the following analyses.

Social desirability was assessed using the shortened social desirability scale. Six items were randomly sampled from the original social desirability scale (Schuessler et al., 1978). One sample item is “I find that I can help others in many ways.” Participants rated each item on a 6-point Likert scale (1 = *strongly disagree*, 6 = *strongly agree*), and higher scores represented a higher level of social desirability. The Cronbach’s α was 0.72.

### Results

#### Preliminary Analyses

Table 1 presents the means, standard deviations and correlations of all variables. In keeping with Hypothesis 1, trait awe is negatively related with social dominance orientation (*r* = -0.38, *p* < 0.001), and positively associated with ecological behavior (*r* = 0.41, *p* < 0.001). Additionally, social dominance orientation is negatively related to ecological behavior (*r* = -0.41, *p* < 0.001).

**Table 1:** Descriptive statistics and correlations between measured variables.

Variables	*M*	*SD*	1	2	3	4	5	6	7
(1) Gender	0.57	0.50	1						
(2) Age	29.55	8.89	0.06	1					
(3) Education	1.73	0.87	-0.03	0.08	1				
(4) Social desirability	3.99	0.69	0.15***	0.09*	0.08	1			
(5) Trait awe	3.93	0.43	0.04	0.04	0.10*	0.10*	1		
(6) Social dominance orientation	3.04	0.94	-0.08	-0.04	-0.05	-0.08	-0.38***	1	
(7) Ecological behavior	3.43	0.67	0.08	0.01	0.11*	0.09*	0.41***	-0.41^∗∗∗^	1


*Gender was dummy coded as 0 = male and 1 = female. Education was coded as 0 = high school education or less, 1 = college degree, 2 = bachelor degree and 3 = post-graduate degree. ^∗^*p* < 0.05; ^∗∗∗^*p* < 0.001.*

#### The Effect of Awe on Ecological Behavior

We examined Hypothesis 1 that awe positively predicts ecological behavior. The control variables were inputted, followed by trait awe, into a hierarchical regression analysis. The results showed that trait awe (β = 0.40, *SE* = 0.04, *F*(5,523) = 23.30, *p* < 0.001, 95% CI [0.32, 0.48]) is positively related to ecological behavior. Thus, Hypothesis 1 is supported.

#### Mediation via Social Dominance Orientation

**FIGURE 1:**
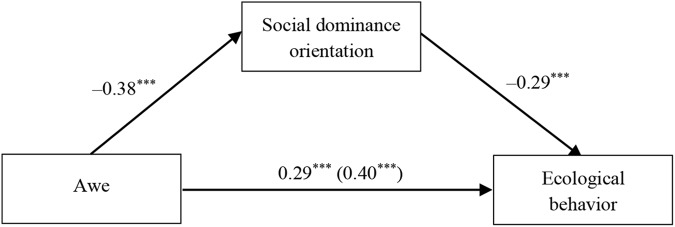
Mediation model for Study 1. ^∗∗∗^*p* < 0.001.

We tested whether social dominance orientation mediates the effect of trait awe on ecological behavior. Model 4 of Hayes’ PROCESS (*N* = 5000) was utilized (Hayes, 2013). As illustrated in Figure 1, after adjusting for the control variables, the results lend credence to Hypothesis 3 that the link between trait awe and ecological behavior is mediated by social dominance orientation (β_indirect_= 0.11, *SE* = 0.02, *F*(6,522) = 29.84, *p* < 0.001, 95% CI [0.08, 0.15]). Hypothesis 2 is thus verified.

### Discussion

These results support Hypotheses 1 and 2, suggesting that individuals’ trait tendencies to experience awe affect their social dominance orientation, which in turn partially mediates the effects of trait awe on ecological behavior. However, the correlational nature of Study 1 constrains the interpretability. Therefore, in Studies 2 to 3, we experimentally manipulated awe to test its causal effects on environmentalism and examine the mediation model proposed in Hypothesis 2.

## Study 2 Causal Research

Study 2 was also has two objectives. First, this study examined the causal relationship between awe and environmental sacrifice and determined whether feeling awe, relative to feeling happiness, increases participants’ willingness to make personal sacrifices for the environment. Second, we attempted to replicate the mediation effect of social dominance orientation on the relationship between awe and environmentalism. We selected happiness as the comparison emotion because both emotions are positive and can broaden individuals’ perspective (Fredrickson, 2001), but they differ in whether perceptual vastness and need for accommodation are experienced or not (Shiota et al., 2007). Moreover, happiness has been used as a positive emotion with which to contrast the effects of awe in previous research (Rudd et al., 2012; Jiang et al., 2018).

### Methods

#### Participants

There were 179 Chinese adults were recruited via Qualtrics to complete an online survey in exchange for monetary compensation. The final valid sample comprised 168 participants (*M*_age_ = 23.57 years, *SD* = 4.67 years; 115 female and 53 male), and 11 participants were excluded from the analysis for failing to complete the manipulation correctly (i.e., wrote something unrelated to experiencing the corresponding emotion). Participants varied considerably in terms of education levels (0.60% with high school education or less, 13.70% with a college degree, 70.20% with a bachelor degree, and 15.50% with a post-graduate degree).

#### Procedure

All participants were required to sign an informed consent form prior to the study, and all procedures were ensured approval by the ethics board. This study includes four parts. First, a between-subject design was adopted, and participants were randomly assigned to one of three narrative recall conditions, namely, awe condition (*N* = 57), happiness condition (*N* = 55), and neutral condition (*N* = 56). In each condition, participants were asked to recall and describe a narrative regarding a personal experience that is an elicitor of the corresponding emotion. This method has been proven to be a well-validated priming technique to induce specific emotions (Piff et al., 2015). The specific instructions were as follows (adopted from Piff et al., 2015; Bai et al., 2017).

##### Awe condition

When experiencing awe, people usually feel like they are in the presence of something so great that their current understanding of the world, their surroundings, or themselves is challenged in some way. Please think about a particular time, fairly recently, when you encountered a natural scene that made you feel awe. This might have been a glorious sunset, a magnificent landscape, or any other time you were in a natural setting that you felt was amazing.

##### Happiness condition

When experiencing happiness, people usually feel delighted by something that satisfies their inner needs and desires. Please think about a particular time, fairly recently, when you felt happy. This might have been attending a birthday party, joining a happy family party, having a nice time with friends, or any other time you encountered something that made you feel happy.

##### Neutral condition

Please take a few minutes to think about something you did fairly recently. This might have been riding a bike, studying for a test, or any other thing that happened during your day.

All participants in each condition were asked to write at least eight sentences (at least 100 words) describing their experiences: what happened, when it happened, who was involved, what they saw, and the accompanying emotions and thoughts. In a post-study review of the written sentences, all participants were ensured to follow the instructions.

Second, participants were required to report their current emotion states and the accompanying sense of self-diminishment.

Third, to reduce participants’ potential demand characteristics, they were asked to complete unrelated items pertaining to their attitudes about sport and entertainment news consisting of a filler task.

Lastly, participants completed measures of social dominance orientation, environmental sacrifice, and demographic information in sequence. Upon completion, they were thanked and debriefed.

#### Measures

##### Current emotion state

Participants reported the degree to which they currently felt each of seven emotions using single items (1 = *not at all*, 7 = *extremely*): anger, disgust, sadness, fear, pride, happiness, and awe (Piff et al., 2015).

##### Social dominance orientation

Six items were used in this study to access participants’ social dominance orientation. The short four-item version of the social dominance orientation scale was adopted to evaluate participants’ social dominance orientation in a general sense (Pratto et al., 2013). One sample item is “Group equality should be our ideal.” Additionally, two items about human dominance over nature were also adopted in our specific setting (Milfont and Duckitt, 2010). One sample item is “Human beings were created or evolved to dominate the rest of nature.” Each item was rated on a seven-point Likert scale (1 = *strongly disagree*, 7 = *strongly agree*), with higher scores indicating higher levels of social dominance orientation. The Cronbach’s α was 0.73.

##### Environmental Sacrifice

Two environmental sacrifice items were used to assess the willingness of participants to make self-sacrifices for environmental protection. The items were “are you willing to make sacrifices to your standard of living (e.g., accept higher prices, drive less, and conserve energy) to protect the natural environment?” and “are you willing to change your daily routine to protect the environment?” (Liu and Sibley, 2012). Participants rated these items on a seven-point Likert scale (1 = *definitely no*, 7 = *definitely yes*). The Cronbach’s α was 0.81.

##### Control variables

In addition to gender, age, and education, we also included self-diminishment as control variable that potentially influenced the relationship between awe and pro-sociality (Piff et al., 2015), so as to clarify the mediating role of social dominance orientation in the relationship between awe and environmentalism in the following analyses.

Self-diminishment was measured with one item (i.e., I feel small or insignificant) taken from Piff et al. (2015). Participants rated this item on a seven-point Likert scale (1 = *strongly disagree*, 7 = *strongly agree*), and higher scores represented a higher level of self-diminishment.

### Results

#### Manipulation Check

We conducted a multivariate analysis of variance to access the effectiveness of emotion priming manipulation. The results demonstrated that the three groups varied in terms of awe, *F*(2,165) = 64.69, *p* < 0.001, $\eta_{p}^{2}$ = 0.44, and happiness, *F*(2,165) = 51.15, *p* < 0.001, $\eta_{p}^{2}$ = 0.38. Participants in the awe condition (*M* = 5.26, *SD* = 1.04) reported higher levels of awe than those in the happiness (*M* = 3.29, *SD* = 1.29) and neutral conditions (*M* = 3.04, *SD* = 1.08; awe vs. happiness: 95% CI for mean difference [1.55, 2.40], *p* < 0.001; awe vs. neutral: 95% CI for mean difference [1.80, 2.65], *p* < 0.001), whereas participants in the happiness condition (*M* = 5.27, *SD* = 1.21) reported higher levels of happiness than those in the awe (*M* = 3.40, *SD* = 0.92) and neutral conditions (*M* = 3.09, *SD* = 1.49; happiness vs. awe: 95% CI for mean difference [1.41, 2.33], *p* < 0.001; happiness vs. neutral: 95% CI for mean difference [1.72, 2.64], *p* < 0.001). No differences were observed in anger, disgust, sadness, fear, or pride across the conditions (*ps >* 0.14, $\eta_{p}^{2}$ < 0.023). These results suggests that our manipulation of the target emotions was successful.

#### The Effect of Awe on Environmental Sacrifice

A one-way analysis of variance (ANOVA) demonstrated that a significant difference in environmental sacrifice across the three conditions, *F*(2,165) = 16.56, *p* < 0.001, $\eta_{p}^{2}$ = 0.17. *Post hoc* analysis revealed that participants in the awe condition (*M* = 5.65, *SD* = 0.96) reported higher levels of environmental sacrifice than those in the happiness (*M* = 5.01, *SD* = 0.85) and neutral conditions (*M* = 4.74, *SD* = 0.76; awe vs. happiness: 95% CI for mean difference [0.32, 0.96], *p* < 0.001; awe vs. neutral: 95% CI for mean difference [0.59, 1.23], *p* < 0.001); the difference between happiness and neutral conditions was not significant (95% CI for mean difference [-0.06, 0.59], *p* = 0.10). As expected, feeling awe increased participants’ willingness to make self-sacrifices for the environment, but feeling happiness did not. Therefore, Hypothesis 1 is supported.

#### The Effect of Awe on Social Dominance Orientation

A one-way ANOVA showed that there was a significant main effect for emotion manipulations on social dominance orientation, *F*(2,165) = 9.07, *p* < 0.001, $\eta_{p}^{2}$ = 0.10. *Post hoc* analysis revealed that participants in the awe condition (*M* = 2.86, *SD* = 0.58) reported lower levels of social dominance orientation than those in the happiness (*M* = 3.33, *SD* = 0.87) and neutral conditions (*M* = 3.45, *SD* = 0.86; awe vs. happiness: 95% CI for mean difference [-0.76, -0.18], *p* < 0.01; awe vs. neutral: 95% CI for mean difference [-0.88, -0.30], *p* < 0.001); the difference between happiness and neutral conditions was not significant (95% CI for mean difference [-0.40, 0.18], *p* = 0.45). Also as expected, feeling awe reduced participants’ social dominance orientation.

#### Mediation via Social Dominance Orientation

**FIGURE 2:**
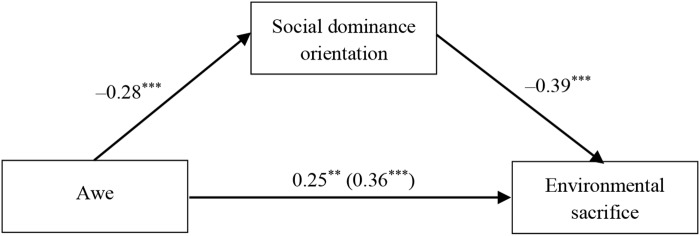
Mediation model for Study 2. ^∗∗^*p* < 0.01; ^∗∗∗^*p* < 0.001.

As reported above, the awe condition led to significant increments in environmental sacrifice and decrements in social dominance orientation. Social dominance orientation was negatively related to environmental sacrifice, *r* = -0.46, *p* < 0.001. Therefore, a mediation analysis was conducted to test whether the awe induction increased participants’ willingness to sacrifice for the environment through reduced social dominance orientation. Model 4 of Hayes’ PROCESS (*N* = 5000) was also employed (Hayes, 2013). As illustrated in Figure 2, after controlling for gender, age, education, and self-diminishment, the positive association between awe and environmental sacrifice is reduced significantly when social dominance orientation is included in the model. Bootstrapping results indicate that the link between awe and environmental sacrifice is mediated by social dominance orientation (β_indirect_= 0.11, *SE* = 0.04, *F*(6,161) = 10.15, *p* < 0.001, 95% CI [0.05, 0.20]). Thus, Hypothesis 2 is verified. These findings are similar to those of Study 1.

### Discussion

Study 2 provided the first experimental piece of evidence for the prediction that awe is positively associated with environmental sacrifice. Recalling a time when participants experienced awe, relative to a happiness or a neutral condition, decreased their social dominance orientation, which in turn increased their willingness to make self-sacrifices for the environment. The findings of Study 2 are similar to those of Study 1.

However, several limitations exist. First, Study 2 depended on participants’ retrospective self-reports, which may reflect their memories of the events but not the awe experience itself (Piff et al., 2015), and this may have influenced their subsequent attitudes. Therefore, Study 3 was designed to broaden and expand upon our previous mediational results by using more tightly controlled experiments in the laboratory. Second, previous research has indicated that nature exposure may influence people’s environmental behaviors (Zelenski et al., 2015). However, our manipulation of awe in Study 2 primarily focused on the nature elicitor, which may corrupt the specificity effect of awe to a certain extent. Meanwhile, other elicitors of awe aside from nature were also present (Keltner and Haidt, 2003). The idea that the awe experienced in non-nature environments can also increase environmentalism remains unexamined. In light of these concerns, Study 3 was conducted to further clarify the independent effect of awe on environmentalism and ascertain whether nonnature-based awe (e.g., social elicitors) triggers environmentalism or not.

## Study 3 Causal Research

In Study 3, awe was induced by exposing participants to awe-inspiring video clips. In addition to a nature-based awe condition (i.e., appreciation of extraordinary nature scenes), we chose mundane nature (e.g., grass) as a comparison condition to help us rule out the possibility that the effect of awe on environmentalism is reducible to mere nature exposure. Mundane natural environments are probably among the most familiar types of nature for people (Joye and Bolderdijk, 2015). Moreover, we verified the generalizability of our findings by incorporating an awe experience induced by social elicitors (i.e., wonder at childbirth). The fourth video was neutral and acted as the control. These conditions allowed us to further test whether awe increases environmentalism and determine whether social dominance orientation mediates the effect of awe on environmentalism.

### Methods

#### Participants

Participants were 187 from a public university in Shanghai, China. The final valid sample comprised 174 students (*M*_age_ = 21.71 years, *SD* = 2.32 years; 105 female and 69 male), and 13 participants were excluded from the analysis: seven for failing to complete the questionnaires (i.e., substantial missing data), and six for failing to complete the manipulation correctly (i.e., wrote something unrelated to the corresponding video). Participants varied considerably in terms of majors, such as law, sociology, psychology, and management.

#### Procedure

All participants were required to sign a written informed consent form prior to the study, and all procedures were ensured approval by the ethics board. This study includes four parts. First, participants were seated in front of computers in private cubicles, asked to put on headphones, and were randomly assigned to one of four video clip conditions: social awe condition (*n* = 44), natural awe condition (*n* = 45), mundane nature condition (*n* = 43), and neutral condition (*n* = 42). The video clips have been validated in previous research (Saroglou et al., 2008; Piff et al., 2015; Davis, 2016).

##### Social awe condition

A 5-min childbirth video clip depicting the image of the fetus on a sonogram and the birth of the baby in a maternity hospital, followed by the mother holding her infant in the first minutes after childbirth.

##### Natural awe condition

A 5-min nature video clip from the BBC’s Planet Earth series, composed primarily of mountains, waterfalls, oceans, forests, deserts, space, and canyons, accompanied by uplifting music.

##### Mundane nature condition

A 5-min nature video clip depicting the grass swaying in the wind, accompanied by nature sounds (crickets chirping).

##### Neutral condition

A 5-min neutral video clip depicting an individual introducing each stage of beer brewing.

After watching the corresponding video, participants were required to write at least five sentences describing the video content and summarizing its gist. They were then asked if there was anything else the video would like to tell us. The description not only can help to enhance the priming effect, but also can be used to check whether participants carefully watched the experiment video or not.

Second, participants were asked to complete the questions on current emotion states and the sense of self-diminishment used in Study 2 to check the specific states induced by the video clips.

Third, participants completed a distracting task to minimize potential demand characteristics. They were requested to search for 10 hidden neutral words in grids of letters (Prade and Saroglou, 2016).

Lastly, participants completed measures of social dominance orientation, pro-environmental intention, and demographic information in sequence. Upon completion, they were thanked and debriefed.

#### Measures

##### Current emotion state

Participants’ current emotion state was assessed using the seven feeling single items as in Study 2 (Piff et al., 2015).

##### Social dominance orientation

Social dominance orientation was assessed as in Study 2. The Cronbach’s α was 0.74.

##### Pro-environmental intention

Pro-environmental intention was assessed by asking how likely it is that participants would buy organic local food, buy less non-essential stuff, buy fewer new things, recycle things, and eat fewer meat meals in the future (Fielding and Head, 2012). All items were rated on a seven-point Likert scale (1 = *very unlikely*, 7 = *very likely*), with higher scores indicating higher levels of pro-environmental intention. The Cronbach’s α was 0.79.

##### Self-diminishment

Self-diminishment was assessed as in Study 2.

### Results

#### Manipulation Check

Similar to Study 2, multivariate ANOVA (MANOVA) was conducted to further test the effectivity of emotion priming manipulation. The awe priming manipulation was successful, *F*(3,170) = 49.90, *p* < 0.001, $\eta_{p}^{2}$ = 0.47. Participants in the social (*M* = 5.70, *SD* = 1.09) and natural (*M* = 5.42, *SD* = 1.16) awe conditions reported higher levels of awe than those in the mundane nature condition (*M* = 3.35, *SD* = 1.29; social awe vs. mundane nature: 95% CI for mean difference [1.83, 2.88], *p* < 0.001; natural awe vs. mundane nature: 95% CI for mean difference [1.55, 2.59], *p* < 0.001) and neutral condition (*M* = 3.21, *SD* = 1.39; social awe vs. neutral: 95% CI for mean difference [1.96, 3.02], *p* < 0.001; natural awe vs. neutral: 95% CI for mean difference [1.69, 2.73], *p* < 0.001). The social and natural awe conditions produced similarly high levels of awe (95% CI for mean difference [-0.23, 0.80], *p* = 0.28), and the difference between mundane nature and neutral conditions was not significant (95% CI for mean difference [-0.39, 0.66], *p* = 0.62). Furthermore, the ratings of other emotions, such as anger, disgust, sadness, fear, pride, or happiness did not differ across conditions (*ps >* 0.25, $\eta_{p}^{2}$ < 0.024). These results suggest that our manipulation effectively evoked the target emotion.

#### The Effect of Awe on Pro-environmental Intention

A one-way ANOVA showed that there was a significant main effect for emotion manipulations on participants’ pro-environmental intention, *F*(3,170) = 8.93, *p* < 0.001, $\eta_{p}^{2}$ = 0.14. *Post hoc* analysis revealed that participants’ pro-environmental intention was higher in the social (*M* = 5.70, *SD* = 0.73) and natural (*M* = 5.60, *SD* = 0.75) awe conditions compared with the mundane nature condition (*M* = 5.02, *SD* = 0.90; social awe vs. mundane nature: 95% CI for mean difference [0.31, 1.04], *p* < 0.001; natural awe vs. mundane nature: 95% CI for mean difference [0.21, 0.94], *p* < 0.01) and neutral condition (*M* = 4.92, *SD* = 1.06; social awe vs. neutral: 95% CI for mean difference [0.40, 1.14], *p* < 0.001; natural awe vs. neutral: 95% CI for mean difference [0.31, 1.04], *p* < 0.001). In addition, the difference between social and natural awe conditions (95% CI for mean difference [-0.27, 0.46], *p* = 0.60), and that between mundane nature and neutral conditions (95% CI for mean difference [-0.27, 0.47], *p* = 0.60) were both not significant. As expected, feeling awe increased participants’ pro-environmental intention. Therefore, Hypothesis 1 is again supported.

#### The Effect of Awe on Social Dominance Orientation

A one-way ANOVA demonstrated that the three types of emotion priming exerted a significant effect on social dominance orientation, *F*(3,170) = 4.47, *p* < 0.01, $\eta_{p}^{2}$ = 0.07. *Post hoc* analysis revealed that participants’ social dominance orientation was lower in the social (*M* = 2.73, *SD* = 0.91) and natural (*M* = 2.69, *SD* = 0.96) awe conditions compared with the mundane nature condition (*M* = 3.17, *SD* = 0.72; social awe vs. mundane nature: 95% CI for mean difference [-0.83, -0.06], *p* < 0.05; natural awe vs. mundane nature: 95% CI for mean difference [-0.86, -0.09], *p* < 0.05) and neutral condition (*M* = 3.26, *SD* = 1.03; social awe vs. neutral: 95% CI for mean difference [-0.92, -0.14], *p* < 0.01; natural awe vs. neutral: 95% CI for mean difference [-0.95, -0.18], *p* < 0.01). In addition, the difference between social and natural awe conditions (95% CI for mean difference [-0.35, 0.42], *p* = 0.86), and that between mundane nature and neutral conditions (95% CI for mean difference [-0.48, 0.30], *p* = 0.66) were both not significant. Also as expected, feeling awe reduced participants’ social dominance orientation.

#### Mediation via Social Dominance Orientation

**FIGURE 3:**
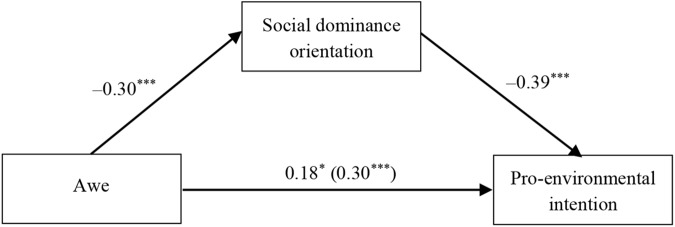
Mediation model for Study 3. ^∗^*p* < 0.05; ^∗∗∗^*p* < 0.001.

As reported above, the experience of awe led to lower levels of social dominance orientation and greater levels of pro-environmental intention compared with the mundane nature and neutral condition. Furthermore, social dominance orientation was negatively associated with participants’ pro-environmental intention, *r* = -0.46, *p* < 0.001. Thus, a mediation analysis was conducted to examine whether the awe conditions influenced participants’ pro-environmental intention through social dominance orientation. Model 4 of Hayes’ PROCESS (*N* = 5000) was also utilized (Hayes, 2013). As in Study 2, we clarified the mediating role of social dominance orientation by controlling for gender, age, and self-diminishment. As illustrated in Figure 3, the positive association between awe and pro-environmental intention is reduced significantly when social dominance orientation is included in the model. Bootstrapping results indicate that the link between awe and pro-environmental intention is mediated by social dominance orientation (β_indirect_ = 0.12, *SE* = 0.04, *F*(5,168) = 14.04, *p* < 0.001, 95% CI [0.05, 0.21]). Thus, Hypothesis 2 is again verified.

### Discussion

The findings of Study 3 advance our understanding of the relationship between awe and environmentalism in several ways. First, eliciting awe using awe-inspiring natural scenes increased participants’ pro-environmental intention in contrast to mundane nature and neutral condition. This helps confirm the unique effects of awe on environmentalism and rule out the influence of mere nature exposure. Second, eliciting awe using nature-based or social elicitors similarly enhanced participants’ pro-environmental intention, indicating that the effect of awe on environmentalism is not limited to experiences in extraordinary nature scenes. Moreover, the awe conditions also lowered participants’ social dominance orientation, which partially mediated the effect of awe on environmentalism. Taken together, these findings lend support to our two hypotheses.

## General Discussion

The current study extends preliminary research on environmental behavior by providing correlational and experimental evidence to explore the relationship between awe and environmentalism. More importantly, we examined why awe enhances environmentalism. Across the three sub-studies, our investigation yielded consistent evidence that awe encourages environmentalism partially because it can reduce individuals’ social dominance orientation. Awe ameliorates the pervading belief in human hierarchical dominance over nature, which in turn increases the likelihood to act on environmental issues.

The results of the three sub-studies indicated that awe positively predicts environmentalism, which is in line with Hypothesis 1. Specifically, in Study 1, individuals with a stronger awe disposition demonstrated more ecological behaviors. In Study 2, participants who recalled an awe experience reported higher willingness to make self-sacrifices for the environment than their counterparts in the happiness and neutral conditions. The effect on environmental sacrifice was specific to awe and was not the result of happiness. This result is highly consistent with those of prior research indicating that the moral consequences of awe are specific to awe and not the effect of other positive emotions, such as amusement (Rudd et al., 2012; Piff et al., 2015). In Study 3, compared to mundane nature and neutral condition, awe experiences elicited by nature-based or social stimuli both increased participants’ pro-environmental intention. The significant effect of awe on environmentalism induced by the childbirth video allowed us to generalize our findings to nonnature-based awe experiences to some extent. These results are in accordance with previous evidence reporting a positive relationship between awe and prosocial behaviors (Rudd et al., 2012; Piff et al., 2015; Prade and Saroglou, 2016), and highlight that awe can broadly influence environmentalism.

The effect of awe on environmentalism was partially explained by social dominance orientation and thus supports Hypothesis 2. The experimentally induced awe decreased individuals’ social dominance orientation, which in turn encouraged the individuals to express high willingness to sacrifice for the environment (Study 2) and exhibit high intentions to engage in pro-environmental behaviors (Study 3). Awe induction weakened individuals’ views of human dominance over nature, which indicates a relative diminishment of the sense of entitlement and superiority, paralleling prior research (Piff et al., 2015; Stellar et al., 2017, 2018). Moreover, the lower the individuals’ social dominance orientation was, the higher they endorsed environmentalism. These findings dovetail with prior empirical work showing that social dominance orientation is a reliable negative predictor of environmentalism (Milfont et al., 2013, 2017). Awe is a self-transcendent emotion that can decrease individuals’ sense of superiority and importance, and shift their focus away from personal interests toward the concerns of others and the broader natural environment (Piff et al., 2015; Stellar et al., 2017, 2018). Humans always believe that they can dominate over nature; however, awe experiences make them realize the smallness and insignificance of the self and the equality between human and nature. In other words, awe can preclude people’s desire to dominate over nature. Consequently, this irrational hierarchical belief is ameliorated, and actions to protect the environment are increased correspondingly. The positive association between awe and environmentalism is rooted in a highly egalitarian view of the world. Taken together, these results suggests that social dominance orientation plays an important role in explaining the relationship between awe and environmentalism. Notably, the present study again provides empirical evidence that social dominance theory can be extended to understand the relations between humans and the natural environment (Milfont et al., 2013, 2017; Milfont and Sibley, 2014; Panno et al., 2017).

### Implications

The present research has several theoretical and practical implications on environmental protection. First, the results concur with the view that awe is a potent predictor of genuine concern for the environment. Our research examined the social function of awe and extended the study of awe to environmentalism. Meanwhile, understanding the role of awe in shaping individuals’ environmental behaviors contributes to scholarly knowledge on the predictors of environmentalism. Second, very few studies have identified social dominance orientation as an underlying psychological mechanism between awe and environmentalism. The mediating role of social dominance orientation provides an insightful explanation of why individuals highly endorse environmentalism after awe is enhanced. The present study not only improved the research on the social dominance theory, but also verified that social dominance theory can explain the relationships among social groups and between humans and natural environments.

At a practical level, apart from seeking macro-level solutions (e.g., sign the international Paris climate agreement) (Hale, 2016), government spare no efforts to encourage people to behave in highly environmentally sustainable ways. However, no one-size-fits-all solution to environmental problems exists. Our study suggests that the elicitation of awe may encourage people to engage in behaviors that protect the environment. For example, in addition to the external stimulus that is characterized by vastness and need for accommodation (Keltner and Haidt, 2003), loving-kindness meditation can also help evoke individuals’ awe experiences (Stell and Farsides, 2016). Moreover, the negative link between social dominance orientation and environmentalism indicates that directing interventions aimed at reducing social dominance orientation is also a useful mean to address environmental issues. For example, in addition to developing a highly egalitarian view of the world through mindfulness training (Panno et al., 2017), building an equal and environmentally oriented society may attenuate the belief of human dominance over nature and may cushion the negative effect of social dominance orientation on environmentalism (Milfont et al., 2017).

### Limitations and Future Directions

This work has several limitations, which could also serve as future research directions. First, although we manipulated the experimental procedures as rigorously as possible, the use of conventional methods for inducing awe in experimental settings (i.e., narrative recall and watching awe-inspiring videos) may limit the external and ecological validity of the study to some extent. Hence, aside from field study, virtual reality may be a promising technique to elicit awe effectively in subsequent research because of its capability to enhance the intensity of emotional states by providing participants with a sense of “presence” (Chirico et al., 2018). Second, our study primarily manipulated positively valenced varieties of awe, although this has been proven true in many experimental studies on awe (e.g., Shiota et al., 2007; Van Cappellen and Saroglou, 2012; Prade and Saroglou, 2016). Nonetheless, the negative experience of awe elicited by threatening stimuli (e.g., tornadoes and volcanoes) was ignored. This situation brings about an interesting question: does negative awe exert a similar effect on promoting environmentalism as that exerted by positive awe? Future research could explore the effect of negatively valenced varieties of awe on various environmental behaviors. Third, social dominance orientation served as a partial mediator in this study, which suggests the existence of other potential mediators (e.g., personal norms). The path from awe to environmentalism may be complex and needs further research. Lastly, the moderator variables between awe and environmentalism should be explored further to uncover the boundary conditions, which may help us elucidate the degree to which awe experiences increase environmentalism.

## Conclusion

The present research contributes to the growing literature on emotions in the field of environmental psychology and enriches the exploration of the social function of awe. The three sub-studies not only clarify the crucial role of awe in enhancing environmentalism, but also support the role of social dominance orientation as a mediator in this relationship. Specifically, this study emphasizes the importance of awe in attenuating people’s desire to dominate over nature. The findings are novel and theoretically and practically insightful, and they create a valuable foundation for future research.

## Author Contributions

HuZ designed the study and wrote the manuscript. HuZ and HeZ acquired and analyzed the data. HeZ, YX, JL, and WH provided instruction and advice for the study. The manuscript was approved by all authors for publication.

## Conflict of Interest Statement

The authors declare that the research was conducted in the absence of any commercial or financial relationships that could be construed as a potential conflict of interest.

## Supplementary Material

The Supplementary Material contains the data used in this study and can be found online at: https://www.frontiersin.org/articles/10.3389/fpsyg.2018.02367/full#supplementary-material
